# Development of Bilateral Heterotopic Ossification After Survival of Life Threatening Purpura Fulminans

**DOI:** 10.7759/cureus.6731

**Published:** 2020-01-21

**Authors:** Mohammed Asif, Kevin M Klifto, Tomer Lagziel, Julie Caffrey

**Affiliations:** 1 Plastic Surgery, The Johns Hopkins University School of Medicine, Baltimore, USA

**Keywords:** amputation, burns, heterotopic ossification, purpura fulminans, splenectomy, streptococcus pneumoniae

## Abstract

Heterotopic ossification has been reported in patients who have undergone traumatic amputations, burn injuries, and total hip arthroplasty; however, the incidence of heterotopic ossification following purpura fulminans has only been reported in one case with unilateral involvement. Here we present a bilateral lower extremity case of heterotopic ossification as sequelae of purpura fulminans.

A 34-year-old male smoker with a past medical history of stab wounds to the chest and abdomen requiring emergent exploratory laparotomy, diaphragmatic repair, and splenectomy 15 years ago presented to the emergency department with a rapid onset of high fevers, chills and myalgia. He did not receive post-splenectomy prophylactic vaccinations for *Streptococcus pneumoniae*, *Neisseria meningitidis*, and *Haemophilus influenzae*. The patient presented clinically in septic shock with disseminated intravascular coagulation. The patient was admitted to the Medical Intensive Care Unit and subsequent workup suggested *Streptococcus pneumoniae* bacteremia. Over the next 48 hours, the patient developed extensive necrosis of the bilateral upper and lower extremities concerning for purpura fulminans. The decision was made to perform a right transradial forearm amputation as well as bilateral transtibial amputations. He tolerated these procedures and was discharged to an inpatient rehabilitation facility. Approximately four months following his bilateral below knee amputations, the patient had difficulty wearing the prosthetics secondary to pain and eventually discontinued use altogether. At home, he continued to ambulate by bearing weight on his knees while wearing kneepads. He continued to report significant tenderness and pain along the bilaterally, below knee amputation stumps. His physical examination was concerning for significant distal bone formation in his bilateral amputation stump sites without evidence of skin breakdown. Intraoperatively, extensive bony formation was found bilaterally within his soleus muscle flaps, concerning for heterotopic ossification. Postoperatively, the patient was refitted for lower extremity prosthetics.

Similar to burns and trauma, the development of heterotopic ossification in patients with purpura fulminans may be directly related to the inflammatory process and amount of tissue damage. In some cases, heterotopic ossification could be caused from daily living activities, so the timing of diagnostic imaging techniques and clinical intervention is crucial.

## Introduction

Purpura fulminans (PF) is a rare but potentially fatal thrombotic disorder that can occur in association with hereditary or acquired deficiencies of protein C and S. It most commonly progresses from an acute inflammatory response to subsequent disseminated intravascular coagulation (DIC). The mortality rate is reported to be up to 50%, with the most common causes of death being due to DIC and multisystem organ failure [1]. The pathogenesis of sepsis-induced PF is not fully understood. 

Previous literature has suggested that the super antigen toxins produced by gram-positive bacteria disturb the coagulant activity of endothelial cells. This triggers the release of proinflammatory cytokines, resulting in widespread capillary thrombosis [2-4]. The most common cause is *Neisseria meningitidis*, followed by *Staphylococcus aureus*, *Streptococcus pneumonia*, and less frequently groups A and B beta-hemolytic *Streptococci*, *Haemophilus influenzae*, and *Escherichia coli* [5-7]. Initial presentation consists of flu-like symptoms (fever, myalgia, and headache), followed by characteristic skin lesions after 12 to 24 hours [8]. Skin lesions begin as well-demarcated erythematous macules that progress rapidly to hemorrhagic necrosis and retiform purpura with vesicle and/or bullae formation. Resultant wet gangrene can develop over the course of hours [9]. The skin necrosis occurs distally in the fingers and toes and progresses proximally [10]. Poor perfusion to distal tissues in the presence of septic shock often leads to multiple limb amputations [11,12].

Late sequelae have been reported in children who developed meningococcal-induced PF. The majority of late sequelae are related to lower limb physeal growth plate arrest and deformities of the knee and ankle [13,14]. Development of heterotopic ossification (HO) has been reported in patients who have undergone traumatic amputations, burn injuries, and total hip arthroplasty; however, the incidence of HO in PF survivors is unknown [15]. There has been one reported case of unilateral right elbow HO following PF [15]. Here we present a bilateral lower extremity case of HO as sequelae of PF. 

## Case presentation

A 34-year-old male smoker presented at the emergency department with a rapid onset of high fevers, chills, and myalgia. His past medical history is significant for stab wounds to the chest and abdomen that required an emergent exploratory laparotomy, a diaphragmatic repair, and a splenectomy 15 years ago. Unfortunately, he did not receive post-splenectomy prophylactic vaccinations for *Streptococcus pneumoniae*, *Neisseria meningitides*, and *Haemophilus influenzae*. His initial mean blood pressure measurements were normal with mean arterial pressure of 70-90 mm Hg with no vasopressors required. His clinical exam was notable for cold, pale palms, and feet. However, serologic workup was remarkable for leukocytosis (47,720/mm^3^), lactic acidosis (12.5 mmol/L), new-onset severe thrombocytopenia (15 K/cu mm), and d-dimer levels > 30.00 mg/L fibrinogen equivalent unit. The patient presented clinically in septic shock with disseminated intravascular coagulation and a petechial rash covering his face, hands, and feet. His metabolic panel indicated decreased protein S activity (47%) and protein C activity in the low end of normal range (74%). After obtaining blood cultures, aggressive fluid resuscitation, vancomycin, and piperacillin/tazobactam were initiated. 

The patient was admitted to the Medical Intensive Care Unit (MICU), and subsequent workup suggested *Streptococcus pneumoniae* bacteremia. Over the next 48 hours (hospital day 2), the patient developed extensive necrosis of the bilateral upper and lower extremities concerning for PF. Furthermore, he developed an acute kidney injury, required vasopressor support (norepinephrine drip, as high as 0.1 mcg/kg/min), and was ultimately intubated for respiratory distress. The treating physicians determined that the patient was likely in a cytokine storm secondary to the *Streptococcus pneumoniae* bacteremia. In the setting of PF with extensive necrotic wounds, the patient was transferred to the Burn Intensive Care Unit (BICU) for further management (hospital day 12). His vitals on BICU admission were notable for temperature 37.3^o^C, blood pressure 136/78 mmHg (on norepinephrine drip, 0.1 mcg/kg/min), heart rate 122 bpm, respiratory rate 17 bpm, and oxygen saturation 97% while on mechanical ventilation. His laboratory data were significant for white count 28,720/mm^3^, hemoglobin 6.5 g/dL, hematocrit 20.4%, blood urea nitrogen 49 mg/dL, creatinine 9.10 mg/dL, and elevated prothrombin time 40.1 seconds.

The patient continued to demonstrate progressive severe necrosis in the affected extremities. He was taken to the operating room for debridement of bilateral upper extremities (hospital day 14 and 17). The bilateral lower extremity wounds were initially managed with local wound care, but severe wet gangrene complicated the situation. Given the significant and persistent hemodynamic instability, operative attempts at limb salvage were considered an unviable option. The decision was made to perform a right transradial forearm amputation (hospital day 23), as well as bilateral transtibial amputations (hospital day 30). He tolerated these procedures and was discharged (hospital day 55) to an inpatient rehabilitation facility on intermittent hemodialysis. 

**Figure 1:**
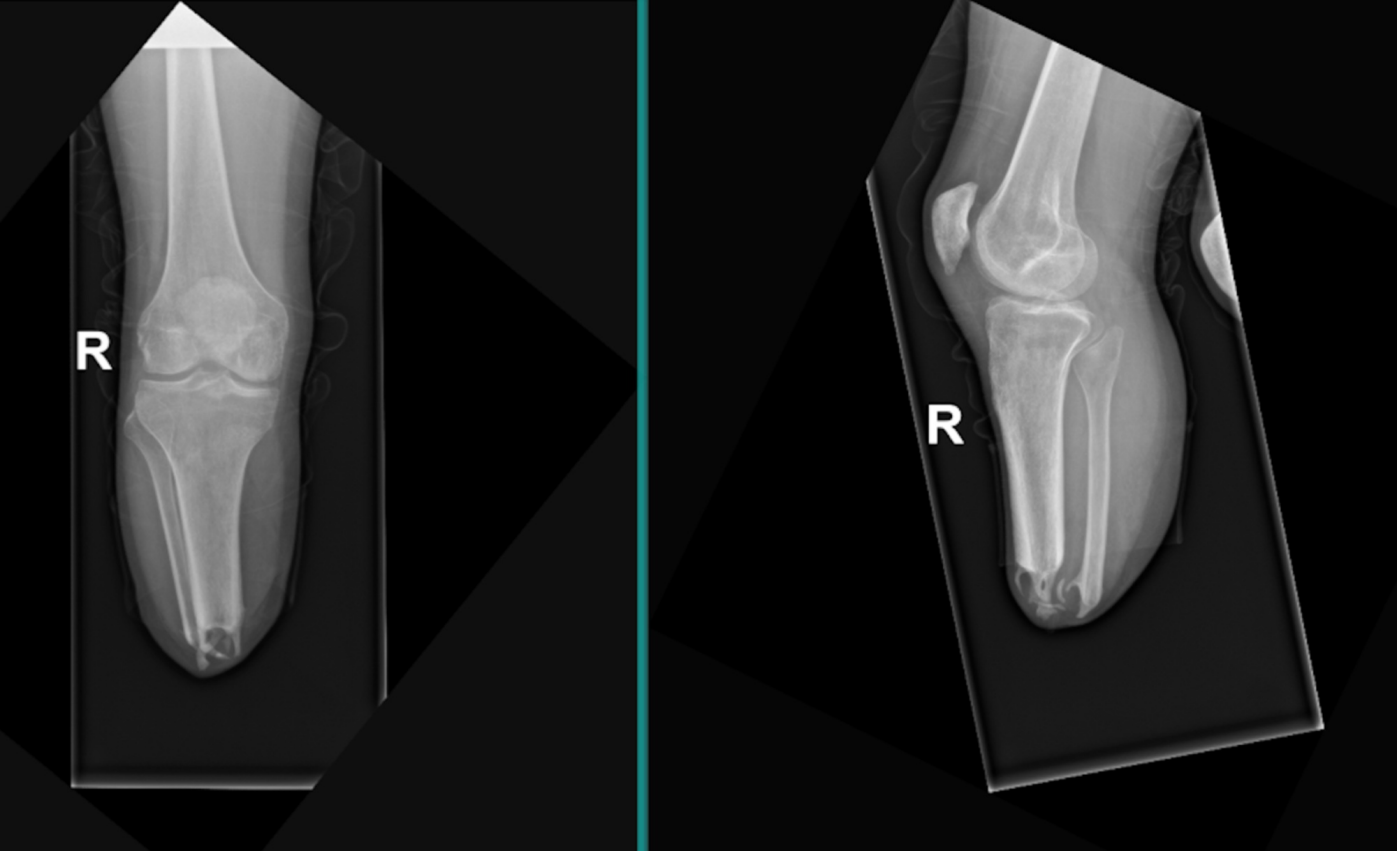
Right lower extremity anteroposterior (left) and lateral (right) plain radiograph of transtibial amputation with distal heterotopic ossification of the tibia and fibula stumps.

**Figure 2:**
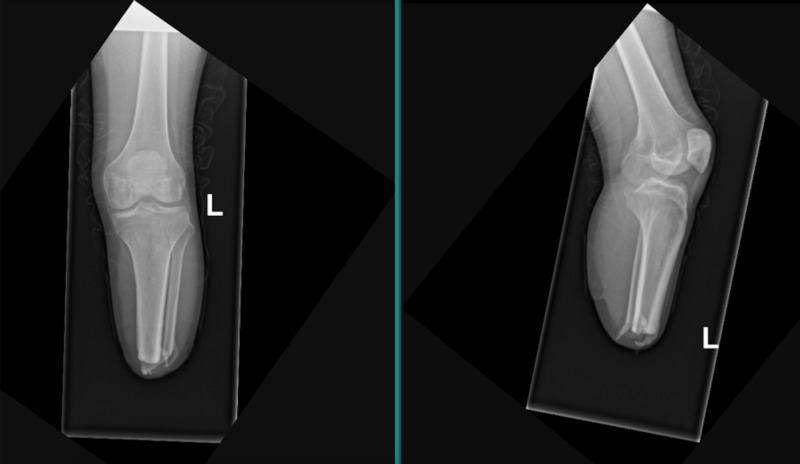
Left lower extremity anteroposterior (left) and lateral (right) plain radiograph of transtibial amputation with distal heterotopic ossification of the tibia and fibula stumps.

While at inpatient rehabilitation, the patient diligently adhered to clinic follow-ups with physical medicine and rehabilitation, burn surgery, and occupational therapy. He was successfully transitioned off of hemodialysis and was fitted for bilateral lower extremity prosthetics at approximately four months following his bilateral below knee amputations (hospital day 142). He had difficulty wearing the prosthetics secondary to pain and eventually discontinued use altogether. At home, during the weeks following being discharged, he continued to ambulate by bearing weight on his knees while wearing kneepads. At his follow-up evaluation appointments, he continued to report significant tenderness and pain along the bilateral below knee amputation stumps. His physical examination was concerning for significant distal bone formation in his bilateral amputation stump sites without evidence of skin breakdown. Radiographic studies were obtained and demonstrated significant bony mineralization extending from the distal most aspect of his bilateral tibiae (Figures 1, 2). He was electively scheduled for an amputation revision of his bilateral lower extremities with a primary focus on excising calcified tissue, indicative of HO. Intraoperatively, extensive bony formation was found bilaterally within his soleus muscle flaps, concerning for HO. Postoperatively, the patient was refitted for lower extremity prosthetics.

**Figure 3:**
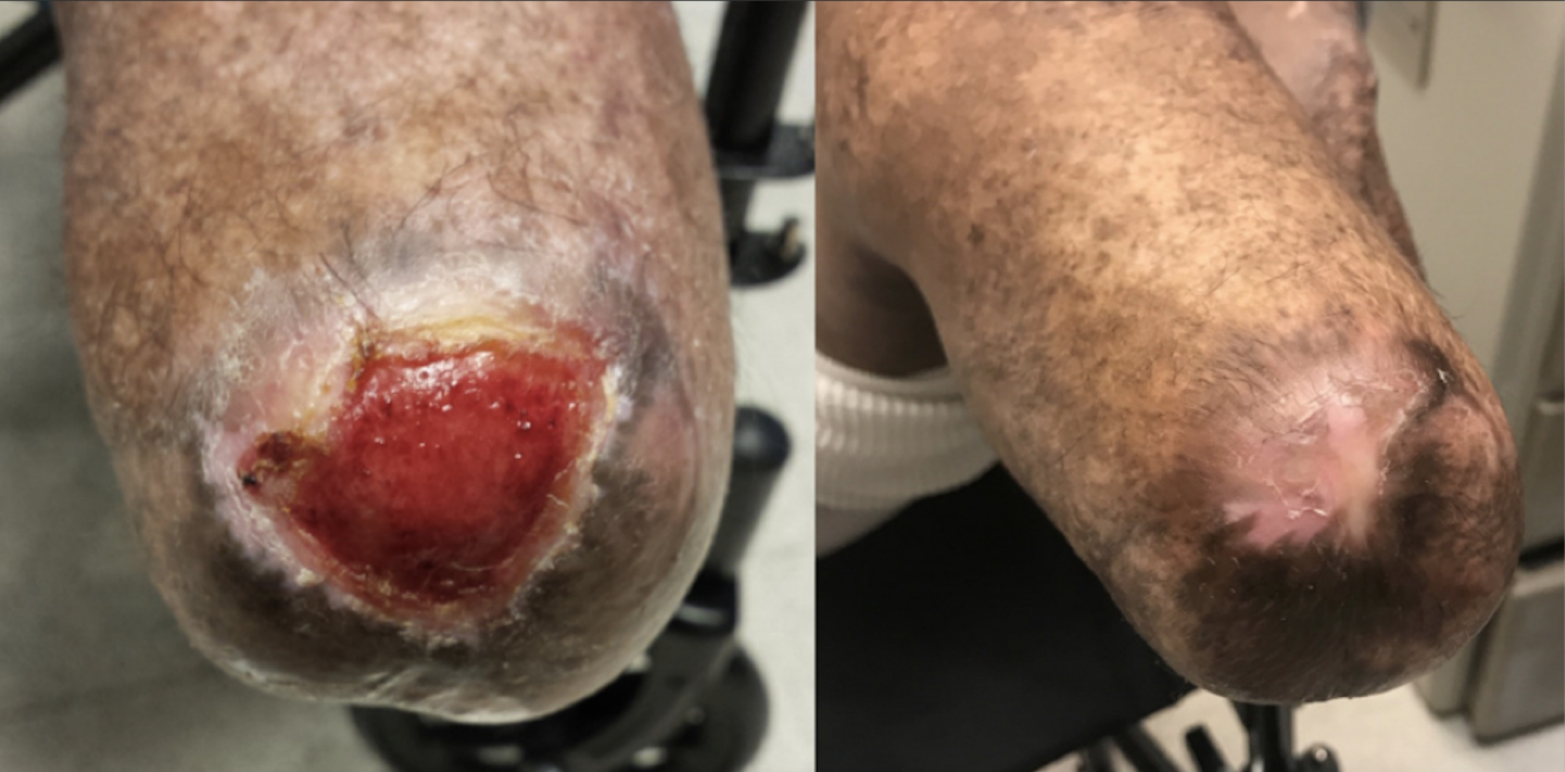
Wound breakdown following left stump revision. Before (left) and after (right) treatment.

Initially, he was able to ambulate with only mild discomfort along the stump sites and transient complaints of phantom pain. One year later, the patient now uses the prosthetics regularly. He partakes in all daily activities including, but not limited to, yard work, playing with his dog, and walking on uneven surfaces as well as up/down stairs. He also developed wounds on his left stump which were debrided and treated topically in the clinic (Figure 3).

## Discussion

HO is the formation of bone in extraskeletal tissues. Dejerine and Ceilier first described it in 1918 in spinal cord injury patients. There are three categories: myositis ossificans progressive, myositis ossificans circumscripita without trauma, and traumatic myositis ossificans. The pathophysiology remains unclear, but it is thought that there is inappropriate differentiation of mesenchymal cells into osteoblast stem cells, similar to physiologic fracture healing [16]. Some risk factors include trauma to the bone or joint space [16].

It is thought that vascular endothelial cells differentiate into skeletal cells through mesenchymal stem cell intermediates. Inflammatory signals at the level of the tissue microenvironment cause differentiation of endothelial-derived mesenchymal stem cells into osteoblasts [16]. An association between a patient’s systemic and local inflammatory response has been linked to HO and wound failure. Acute inflammatory responses have been suggested to induce a cascade event that can lead to HO [17]. In the case of our patient, the cytokine storm led to shock, DIC, and together with the wet gangrenous component of wounds and amputation-related trauma increased the risk for HO. The vast majority of these cases are related to prolonged wounds and delayed closure [18].

As was briefly mentioned above, there has only been one other reported case of unilateral HO in a patient with PF [15]. When comparing that case to our findings, minor similarities can be observed but clear differences are more prominent. Primarily, the previously reported case of HO subsequent to PF was immediately after (during acute inpatient rehabilitation) a unilateral upper extremity amputation. In addition, the previously reported patient developed PF from a *Staphylococcus*-infected intrauterine device as opposed to a burn injury [15]. 

The patient presented in our case started complaining of ambulatory pain four months after his lower extremity amputations. It is unclear whether this pain was associated with HO or if the pain was part of a subacute inflammatory response due to amputation which exacerbated HO. 

Severe trauma, in particular amputation, has been linked to the development of HO as well [18]. When considering amputation in PF, one needs to consider the associated necrotic wet gangrene which significantly increases the need for amputation [19]. Conservative treatment of HO includes physical therapy, but when symptoms become too severe, it should be addressed with surgical excision of ossified tissue [20].

## Conclusions

Similar to cases following trauma and burns, the development of HO in patients with PF may be directly related to the inflammatory process and amount of tissue damage. Not all patients with PF develop HO, but we suggest that it must be considered as part of the pathology when wet-gangrene-induced amputation is involved. This complication greatly increases the risk for HO and should be monitored closely when following up with the patient. Postop patients with similar characteristics to those described in this report should be on the lookout for significant pain or discomfort in the area of prostheses. Imaging techniques should be utilized if clinically necessary. Our management of the patient was consistent with literature findings which call for surgical measures only once conservative treatment options have been exhausted. 
